# Challenges in adjusting scoring matrices when comparing functional motifs with non-standard compositions

**DOI:** 10.1038/s41598-024-82548-8

**Published:** 2024-12-30

**Authors:** Patryk Jarnot

**Affiliations:** Department of Computer Networks and Systems, Silesian University of Technology, 44-100 Gliwice, Poland

**Keywords:** Protein sequence, Non-standard compositions, Similarity search, Scoring matrix adjustment, Protein sequence analyses, Statistical methods

## Abstract

Methods for scoring matrix adjustment decrease the significance of biased residues to better detect homology between protein sequences. This is because non-homologous proteins often contain fragments with non-standard compositions that are strikingly similar to each other. However, these fragments are also functionally important in proteins and are receiving an increasing attention from the scientific community. In this study, we described why the gold standard method for scoring matrix adjustment is unable to emphasise frequent amino acids. Further, we used BLAST to align collagen-like domains with and without the scoring matrix adjustment and compared the results. We found that the scoring matrices were adjusted in the opposite direction to the optimal state. Therefore, turning off the adjustment improved alignment quality of collagen-like domains, but scoring matrices still need refinement. This study provides a detailed analysis of why the gold standard method fails, and opens doors for new methods to adjust scoring matrices for functional motifs with non-standard compositions.

## Introduction

Methods for protein sequence similarity analyses focus on detecting homology in domains with high amino acid diversity. Experimental wet-lab methods for discovering protein properties, while accurate, are slow and expensive. To speed up and reduce cost of whole process, scientists search for similar and well-described proteins to infer a protein of interest and thus reducing a number of experimental scenarios. They scan large protein databases using computational methods, which rely on homology detection between protein sequences with standard amino acid compositions^1^. Consequently, homology detection methods based on these compositions are well developed. Scientists improved them by adding new metrics, scoring matrices and methods for their adjustment to new contexts^2–4^.

On the other hand, fragments with non-standard amino acid compositions have long been ignored by computational biologists, with the result that most of them belong to the dark proteome^5,6^. Motifs characterised by non-standard compositions include homopolymers, short tandem repeats, low complexity regions and compositionally biased regions. They have lower information content than standard protein sequences, thus researchers have initially focused mainly on sequences with standard amino acid compositions. Currently, scientists developed a number of methods to identify them, including CAST, fLPS, SEG, LCD-Composer and SIMPLE^7–11^, but we still lack efficient methods for their similarity analyses. One of the most significant improvements of protein search methods is adjusting scoring matrices to adapt them to different compositional contexts^12–14^. For example, it has been shown that diminishing importance of frequently occurring amino acids improves seraching for domains containing motifs with non-standard compositions^12^. The reason for this is that these regions often occur in proteins as linkers between two functional domains^15^. Therefore, this feature indeed enchances homology detection, but at the same time may prevents efficient analysis of functionally important fragments with non-standard amino acid compositions^16^.

Methods for modifying scoring matrices maintain consistency between target and background frequencies^12,13^. To create a scoring matrix, target frequencies are calculated from multiple sequence alignments of evolutionarily related proteins. Then, background frequencies are described by Eq. (1).

1 $$\begin{aligned} p_i = \sum _{j}q_{ij};\quad \quad p_j = \sum _{i}q_{ij} \end{aligned}$$

where $q_{ij}$ are target frequencies of *i*, *j* amino acid pairs. These frequencies are further used in Eq. (2) to calculate a scoring matrix.

2 $$\begin{aligned} s_{ij} = \frac{1}{\lambda } ln\left( \frac{q_{ij}}{p_ip_j}\right) \end{aligned}$$

where $\lambda$ is a scaling factor that scales the natural logarithm to a logarithm with base used during creation of a particular scoring matrix. Methods for modifying scoring matrices adapt them to different compositional contexts by changing their target and background frequencies. They first compute background frequencies from two sequences, and then adjust target frequencies to satisfy the constraints in Eq. (3).

3 $$\begin{aligned} \sum _{j}Q_{ij} = P_i;\quad \quad \sum _{i}Q_{ij} = P'_j \end{aligned}$$

where $Q_{ij}$ are modified target frequencies for all given amino acid pairs *i*, *j*; $P_i$ and $P'_j$ are background frequencies of given residues of query and database sequences, respectively. Moreover, to improve homology detection they keep the relative entropy of target and background frequencies similar to an original matrix by adding the constraint described in Eq. (4).

4 $$\begin{aligned} h = \sum _{ij}q_{ij}ln\left( \frac{q_{ij}}{p_i p_j}\right) = \sum _{ij}Q_{ij}ln\left( \frac{Q_{ij}}{P_i P'_j}\right) \end{aligned}$$

In order to adjust target frequencies to a new context, these methods use optimisation algorithms such as the Newtonian system. The result is an asymmetric target frequency matrix from which we calculate a scoring matrix using Eq. (5).

5 $$\begin{aligned} S_{ij} = \frac{1}{\lambda } ln\left( \frac{Q_{ij}}{P_iP'_j}\right) \end{aligned}$$

In this study, we have shown that this standard approach for adjusting scoring matrices cannot be used to emphasise frequently occurring residues, and is inefficient for handling functional motifs with non-standard amino acid compositions.

## Methods

In this section, we have demonstrated that methods for recomputing scoring matrices are inefficient in emphasising frequent residues, and we described performed analyses. First, we showed that when comparing two fragments with non-standard amino acid compositions then maximal scores of biased residue matches are unexpectedly low. Therefore, we analysed why this happens by investigating a dependence between background frequency and a maximal score. Next, we described a quantitative analysis that examined the effect of the scoring matrix adjustment in BLAST on a performance of searching for functional motifs with non-standard compositions. For a single alignment, we compared BLOSUM62 with a matrix adjusted using BLAST^4^. Finally, we showed that if we create a new method for scoring matrix adjustment then we also need new metrics for evaluating alignments. The source code utilized in this analysis is available as a package at 10.5281/zenodo.14264780. The package contains python scripts to reproduce the analyses, modified version of BLAST, collagen-like domains from InterPro and UniProtKB/Swiss-Prot sequences^17–19^.

A maximal score on the diagonal in a scoring matrix is inversely proportional to a background frequency. In a symmetric matrix, scores are calculated using Eq. (2). It consists of the $\lambda$ parameter, natural logarithm and ratio of target to background frequencies. The $\lambda$ parameter is a normalising scale factor for different scoring systems^20^. The logarithm scales the ratio to a symmetrical form that clearly indicates whether a particular substitution is likely to occur in homologous sequences or not. The ratio of target to background frequencies is the core of the equation. Target frequencies are divided into 400 pairs of residue types, while background frequencies are divided into 20 types of amino acids. Both types of frequencies sum to 1. Additionally, according to Eq. (1), columns and rows in a target frequency matrix sum to background frequencies of their corresponding amino acids. Note that a maximal score for a given background frequency is obtained by maximising its target frequency in Eq. (2). Hypothetically, if we assume that $q_{ij} = 0$ in all entries of a target frequency matrix where $i \ne j$, then the frequencies on the diagonal reach their highest values, which are equal to their corresponding background frequencies ($q_{ii} = p_i$). Under these assumptions, a score on the diagonal for a symmetric matrix can be calculated using Eq. (6).

6 $$\begin{aligned} s_{ii} = \frac{1}{\lambda } ln\left( \frac{1}{p_i}\right) \end{aligned}$$

Therefore, a maximal score increases when a background frequency decreases, and vice versa.

**Fig. 1:**
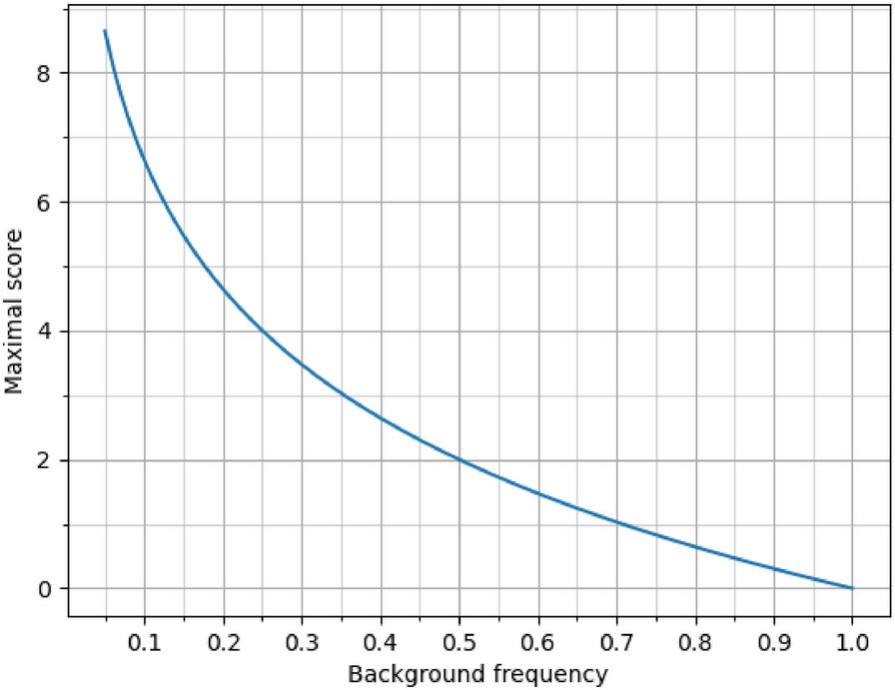
Maximum alignment score decreases as compositional bias of compared sequences increases. Graph plots maximum score for paritcular residue on diagonal by background frequency, assuming that other entries in its row and column are 0.

The standard approach to adjusting scoring matrices is unable to favour frequently occurring residues. In Fig. 1 we demonstrated the relationship between the maximal score and the background frequency using Eq. (6). As the $\lambda$ parameter in the equation we used value *ln*(2)/2 to maintain consistency with the BLOSUM62 scoring matrix. From the figure we can infer that when a background frequency, denoted by the parameter $p_i$, reaches the maximum value of 1, the maximum score $s_{ii}$ is 0. When $p_i$ takes a value of 0.5, the maximum score is 2. Furthermore, as $p_i$ decreases, the maximum score approaches the vertical asymptote at $x = 0$. If we consider the average background frequency (0.05), the maximum score increases to approximately 8.64. Therefore, rarer residues have the potential to achieve higher scores. It is important to acknowledge that in the literature, Eq. (2) is occasionally referred to as the “log-odds”. However, it is essential to recognize that this terminology might not align precisely with the traditional definition of log-odds. Especially in log-odds, a high nominator value automatically leads to a high result value, which differs from the scoring matrix calculation.

Even if we adjust a scoring matrix to emphasise frequent residues by allowing for inconsistencies in amino acid frequencies, we still need to develop new metrics to evaluate results based on the raw alignment score. The e-value and bit score statistics are valid if the expected score of a scoring matrix is negative^20^. Therefore, the matrix must satisfy the condition in Eq. (7).

7 $$\begin{aligned} \sum _{i,j}p_i p_j s_{ij} < 0 \end{aligned}$$

To illustrate, if we modify scoring matrix using a simple approach outlined in Eq. (8), then the expected score may become positive.

8 $$\begin{aligned} S_{ij} = {\left\{ \begin{array}{ll} 2s_{ij}, & \text {if } Q_{ij} >= 0.1\\ s_{ij} / 2, & \text {otherwise} \end{array}\right. } \end{aligned}$$

In practice, when aligning two identical homopolymers of glycine, the expected score of BLOSUM62 adjusted using the outlined approach is equal to 12. In this example we have broken the condition from Eq. (7), thus we should use statistics other than e-value and bit score to evaluate the alignment.

**Fig. 2:**
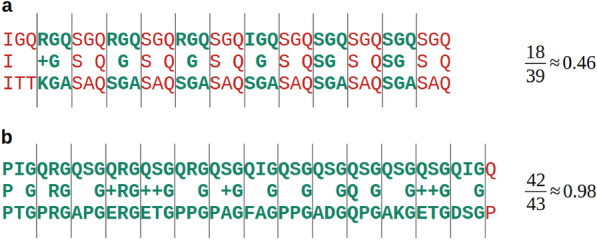
Visualisation of how accuracies of collagen alignments were calculated. Panel (**a**) shows example alignment to non-collagenous motif, while panel (**b**) shows alignment to collagen-like domain.

Next, we quantitatively analysed how the default scoring matrix adjustment in BLAST affects domains with non-standard compositions. To ensure the integrity of the analysis, we sought to avoid poorly studied examples, as this could lead to erroneous speculations regarding the outcome of the results. For this reason, we selected collagen as a well-described example of compositionally biased domains. We used the reviewed collagen repeats from InterPro (version: 100.0) tagged as IPR008160 as a query dataset.^18^. It consisted of 1448 collagen-like domains, which we used twice to search for similarities in UniProtKB/Swiss-Prot (version: 2024_04)^19^. Once using the BLOSUM62 matrix, and once using the default method for adjusting scoring matrices to new contexts. In addition to the scoring matrix adjustment, we also optimised alignment related parameters to achieve the highest true positive rate. To find them, we left the default limit of 250 alignments per query and set an e-value threshold to 10,000,000 in order to maximise the number of alignments per query-higher e-values are impractical as calculations take a long time. As a result, we used the following parameters for searches with scoring matrix adjustment turned off: word size (2), gap open (13) and gap extend (1). For adjusted scoring matrices the values were: word size (4), gap open (9) and gap extend (2). The procedure and results of the parameter optimisation are described in the Supplementary Material in section “Parameter optimisation”. We then calculated an accuracy for each alignment. To calculate this, we divided each midline into triplets and checked whether they all contained a single glycine. If yes, it was marked as a collagen triplet. The accuracy was then calculated as a ratio of the number of amino acids within collagen triplets to a length of an alignment. This procedure is shown visually in Fig. 2. This allowed us to assess the accuracy of collagen-like alignments for the original and modified scoring matrices. We also checked the number of true and false positives in the results. For all alignments, we determined whether a hit protein contained at least one collagen-like domain according to InterPro. If it contained, the alignment was marked as a true positive. The remaining alignments were marked as false positives. To support this study, we also checked whether a corresponding analysis of coiled coil domains lead to the same conclusions. We described the analysis of coiled coils in the Supplementary Material in section “Coiled coil analysis”.

In order to examine how significance of frequently occurring residues might be unexpectedly diminished, we compared BLOSUM62 with this matrix adjusted for collagen-like domains. Therefore, we modified the BLAST source code (version: 2.15.0) to print a target frequency matrix after adjustment. The method modifies matrices based on query and database sequences. In our case, the query was the collagen alpha-1(VII) chain protein (Q02388) at the 1254–2781 sequence position, while the database sequence was the collagen alpha-1(XXVII) chain A protein sequence (C7DZK3). Subsequently, we used the resulting target frequency matrix to calculate an asymmetric scoring matrix using Eq. (5). We compared the modified scoring matrix with the original one to determine whether adjusted matrices are more suitable for identifying the similarity between collagen-like sequences. We then used BLAST with both matrices to align the sequences. In the alignments, we checked if the hit was collagen-like and if it was properly aligned.

We also investigated how BLAST treats fragments whose expected score is theoretically non-negative, and therefore fails to satisfy the requirement from Eq. (7). We used BLAST with default parameters to align two identical homopolymeric sequences. They consisted of prolines and were 20 amino acids long. Such sequences are found in glyceraldehyde-3-phosphate dehydrogenase, testis-specific protein (Q64467) at position 54–73 and in WASP homolog-associated protein with actin, membranes and microtubules (Q8TF30) at position 639–658. We used them with the modified version of BLAST to print out the resulting matrices and analyse the code. In theory, the method could compute only the score for proline match and skip the remaining values as they are useless for aligning the sequences. However, we found that BLAST recalculates the entire matrix regardless of whether a given amino acid pair is used for alignment or not. This can lead to infinite scores in a scoring matrix as a result of division by 0 in all entries where at least one background frequency used for its calculation is 0. In this situation, the method validates the results, and if it encourages the issue, it leaves the Newtonian system with the target frequencies computed in the previous iteration and continues with the scoring matrix calculation. However, to avoid this issue, BLAST combines background frequencies with pseudocounts using a weighted average as in Eqs. (9) and (10).

9 $$\begin{aligned} & {\left\{ \begin{array}{ll} {\bar{P}}_i = (1 - \alpha ) P_i + \alpha p_i\\ \alpha = 20 / (20 + L) \end{array}\right. } \end{aligned}$$

10 $$\begin{aligned} & \quad {\left\{ \begin{array}{ll} {\bar{P}}'_j = (1 - \alpha ') P'_j + \alpha ' p_j\\ \alpha ' = 20 / (20 + L') \end{array}\right. } \end{aligned}$$

where *L* and $L'$ are lengths of query and database sequences, respectively. This ensures that all background frequencies are above 0, and allows all scores to be calculated. The target frequency and scoring matrices computed using BLAST for this case are presented in Supplementary Figs. S1 and S2.

## Results

This section compares the results of the quantitative analysis and the matrices used for the example alignment. First, we showed in general how the gold standard scoring matrix adjustment affects accuracy of collagen-like domain alignments. We then presented the target frequency and scoring matrices adjusted for two protein sequences containing this domain. Finally, we analysed the alignment of two protein sequences calculated using BLAST with both matrices.

### Quantitative analysis of collagen-like domains

Table 1 presents the numbers of alignments grouped by the accuracy intervals calculated when searching for motifs similar to collagen-like domains. From this table we can see that the results obtained using the modified matrices were less accurate than those obtained using BLOSUM62. The average accuracy of the results calculated using the adjustment disabled was about 0.95, while with it enabled the value was about 0.88. Nevertheless, the results obtained with the original matrix still contained inaccuracies. They contained some poorly aligned sequences with the accuracy of less than or equal to 0.1. In this interval, the number of alignments in the results with adjustment turned off was 195, while the number of alignments in the results with adjustment turned on was 60.

We also checked how BLAST performed when similar sequences were classified as collagen-like according to InterPro. In the results calculated with the scoring matrix adjustment, we found about 86.5% of the hits annotated as collagen-like. The value was significantly higher when the adjustment was switched off, and was approximately 94.2%. Turning the scoring matrix adjustment off and on had the greatest effect on the true positive rate. The rate value was about 93.4% for the worst alignment parameters and the BLOSUM62 scoring matrix, which is still about 6.9% higher.

**Table 1:** Adjusting scoring matrix to new context reduces accuracy of collagen-like domain alignments.

Accuracy intervals	Number of alignments by accuracy (original matrix)	Number of alignments by accuracy (modified matrices)
(0.95−1.00>	66,031	39,993
(0.90−0.95>	50,274	56,482
(0.85−0.90>	29,734	41,595
(0.80−0.85>	16,631	32,891
(0.75−0.80>	8,428	25,127
(0.70−0.75>	3,322	18,074
(0.65−0.70>	754	12,567
(0.60−0.65>	205	7,568
(0.55−0.60>	32	4,449
(0.50−0.55>	13	3,008
(0.45−0.50>	6	2,369
(0.40−0.45>	6	2,191
(0.35−0.40>	6	1,735
(0.30−0.35>	4	1,315
(0.25−0.30>	16	935
(0.20−0.25>	4	403
(0.15−0.20>	9	260
(0.10−0.15>	14	78
(0.05−0.10>	30	28
<0.00−0.05>	165	32

Table presents numbers of alignments grouped by accuracy. Alignments were calculated using BLAST with BLOSUM62 matrix and matrices adjusted to new contexts.

### Adjusted target frequency matrix

**Fig. 3:**
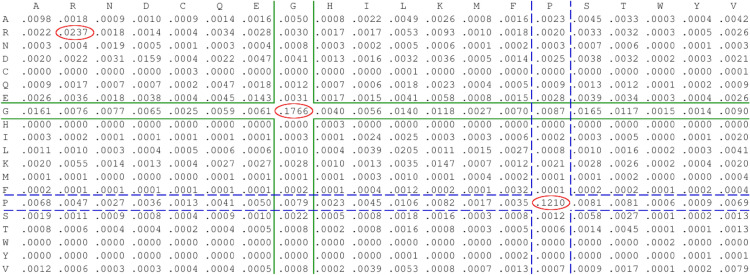
Glycine and proline matches had high values compared to other entries. Target frequency matrix adjusted using background frequencies of collagen-like domain at position 1254–2781 from collagen alpha-1(VII) chain protein (Q02388) as query and whole collagen alpha-1(XXVII) chain A protein (C7DZK3) sequence as database sequence. Source matrix was BLOSUM62. Red circles highlight values which are higher in comparison to source matrix on diagonal. Green line and blue dashed lines indicate glycine and proline mismatches, respectively.

Figure 3 shows the target frequency matrix modified using BLAST. The source matrix was BLOSUM62, which was adjusted to collagen containing background frequencies. The canonical collagen triad (GXY) requires glycine as every third residue, while proline and hydroxyproline often occupy either X or Y residues^21^. Therefore, as expected, the match values for glycine and proline were high, with values of about 0.18 and 0.12, respectively. Interestingly, in addition to glycine and proline, arginine was another residue whose match was more frequent in the adjusted matrix. In the original matrix this match has a frequency value of about 0.0178, while in the adjusted matrix it was approximately 0.0237. This observation is in agreament with research about collagen concluding that arginine at Y position has a beneficial effect on collagen stability^22^. The remaining target frequencies on the diagonal were lower in the adjusted matrix and were less than or equal to 0.0205. For glycine and proline, the values in their rows were higher than these in their column. This is rational, since the entire query was collagen-like, while the database sequence was the protein containing this domain.

### Adjusted scoring matrix

**Fig. 4:**
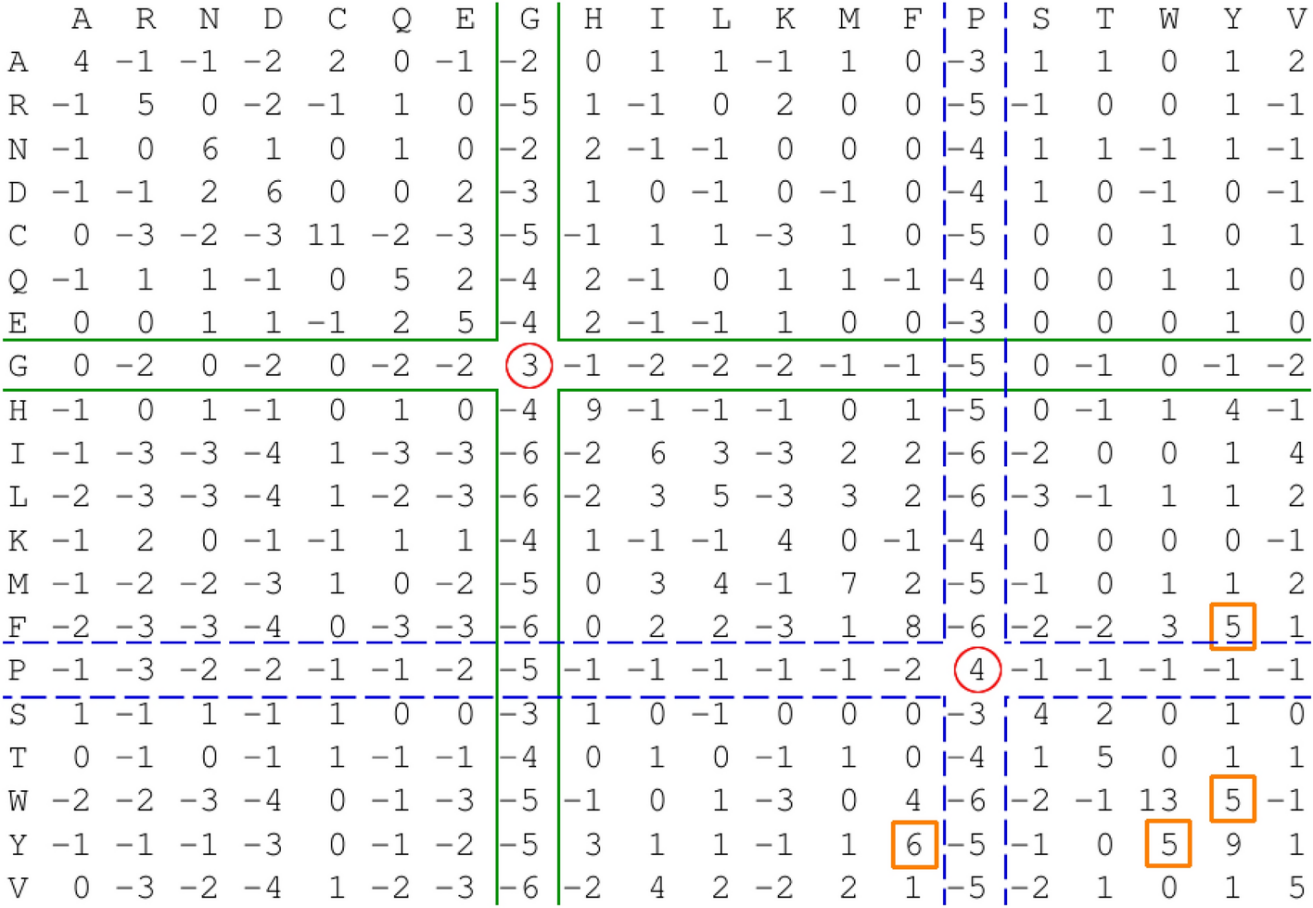
Major residue in collagen-like domains is glycine which has lowest score in adjusted scoring matrix (red circles). Figure presents BLOSUM62 scoring matrix adjusted to collagen-like domain at position 1254–2781 from collagen alpha-1(VII) chain protein (Q02388) as query and whole collagen alpha-1(XXVII) chain A protein (C7DZK3) sequence as database sequence. Orange squares are mismatches with higher values than glycine and proline matches. Green line and blue dashed lines indicate glycine and proline mismatches, respectively.

The method adjusted the scoring matrix, presented in Fig. 4, in the opposite direction to that which would be expected for collagen. Glycine and proline are major residues in collagen, but the scoring matrix adjustment method reduced their scores from 6 to 3 and from 7 to 4, respectively. According to this matrix, the match of tryptophan was over four times more significant than that of glycine. Moreover, the match value for glycine in the modified matrix was lower than these of some mismatches. The highest mismatch value was from tyrosine to phenylalanine with a score of 6, which was even higher than most values on the diagonal. Furthermore, the mismatch scores from phenylalanine to tyrosine and in both directions between tyrosine and tryptophan were 5, which was also high in comparison to the match score of glycine and proline. The method assigned value 4 to six mismatches occurring between tyrosine and histidine, valine and isoleucine, from tryptophan to phenylalanine and from methionine to leucine. In the adjusted matrix, the cost of glycine mutation to other amino acids was on average lower than in the original matrix, falsely suggesting that glycine can be easily replaced with little effect on collagen structure^23^. In BLOSUM62 this mutation is on average about − 2.1, while in the modified matrix it was about − 1.4. On the other hand, mutation costs from other amino acid types to glycine were reasonably higher, with an average score of about − 4.4^24^. A similar phenomenon was observed for proline. For the adjusted matrix, proline mutation was scored on average as about − 1.5 from this residue and − 6.3 to this residue, whereas in the original matrix this value is approximately − 2.1. In the original BLOSUM62 matrix, mutations in both directions have the same average score since the matrix is symmetric.

### Matrix adjustment and alignment accuracy

**Fig. 5:**
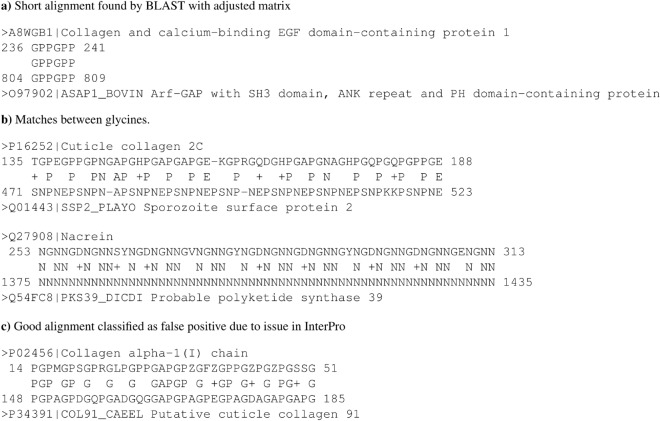
Three types of alignments that cause false positive hits. This figure in (**a**) shows alignment too short to form collagen, in (**b**) aligned sequences due to matches in between glycines, and in (**c**) correct alignment where hit sequence lacks annotation in InterPro. Second alignment in (**b**) was calculated with scoring matrix adjustment disabled while rest of alignments with this parameter enabled.

In practice, an inadequate method for adjusting scoring matrices can lead to inaccurate protein alignments. This is illustrated in Fig. 5, which shows example alignments classified as false positives. The examples demonstrate different types of alignment errors present in both results.

In the first case presented in Fig. 5a, BLAST aligned two short sequences using the adjusted matrix. The length of the query sequence was 40 residues, while the alignment consists of only 6 columns. Such short alignments are frequent when the scoring matrix adjustment is turned on, and they are absent when the adjustment is turned off. The shortest alignment in the results with adjustment disabled consists of 26 columns and there are only three of them. The results with adjustment enabled contain 11,179 alignments shorter than or equal to 26 columns. Such short alignments are reasonable, since for short sequences background frequencies are strongly influenced by pseudocounts, as shown in Eqs. (9) and (10). These sequences of mostly false positives significantly increase the average accuracy in Table 1 and decrease the true positive ratio.

In Table 1 we can see that the results calculated using the original matrix have a high number of alignments with high accuracy, a low number of alignments with the accuracy of about 0.4, and a slightly higher number of alignments with the accuracy less than or equal to 0.1. In the case of adjusted matrices, these numbers change smoothly from high values at the top to low values at the bottom. The reason can be deduced from Fig. 5b which are alignments without any glycine match thus their alignment accuracy is 0. The first example, calculated using the scoring matrix adjustment enabled, contains different residues between glycine matches. Such alignments are common in the results, and introduce a variable number of random glycine mismatches in hits. In the second example, which was calculated with the scoring matrix alignment disabled, the query consists of GNN repeats, which can easily be aligned to poly-N sequences with high scores. Databases contain such homopolymers and they rarely contain glycine mutations, thus there are fewer alignments in the middle of the table than at the end. Other examples of collagen-like triplets forming short tandem repeats are GDK, GSS and GPP, found in proteins with the following UniProtAC:O96614 Q5UNS9 and P22576.

Figure 5c shows well-aligned sequences that are not annotated as collagen-like domains in the InterPro database. This is a common challenge associated with biological data, and such alignments are present in both results. For a given protein lacking a particular annotation, we cannot be sure whether it lacks the property or the annotation^25^.

## Discussion

In this paper, we have analysed how the scoring matrix adjustment of BLAST affects searching for functional motifs with non-standard compositions. In the methods section, we described how significance of frequently occuring residues is diminished by methods for their adjustment, and we described the consequences of emphasising these residues on the use of the e-value and bit score statistics. We also collected results of the quantitative analysis of the collagen-like domains, compared BLOSUM62 before and after modification and analysed the example alignment.

Our findings showed that the scores were adjusted in a direction that was contrary to the optimal state. The default method for adjusting scoring matrices in BLAST decreased the significance of the glycine match when comparing two collagen-like domains. Nevertheless, this residue is of particular importance in collagen as it is required at every third position in a sequence. Furthermore, residues situated between glycines are often prolines and hydroxyprolines, which stabilise the structure of collagen^21^. However, this residue on the diagonal in the adjusted matrix had the second lowest score. As a consequence, pairs of residues irrelevant to collagen had higher scores than relevant pairs. This led to inaccurate alignments as illustrated in the first alignment in Fig. 5b. Our quantitative results, presented in Table 1, also showed that we obtained significantly less inaccuracies in the alighments with the original matrix than with the modified matrices. Glycine mutations in collagen have been shown to be responsible for human disease to varying degrees, depending on the physicochemical similarity of the target amino acid^26^. The analysis of true and false positives also showed a significantly better performance of BLAST when the scoring matrix adjustment was turned off. In the analysis of coiled coils described in the Supplementary Material in section “Coiled coil analysis”, we also observed that turning on the scoring matrix adjustment decreased the true positive rate. Therefore, a scoring matrix adjusted by BLAST for domains with non-standard amino acid compositions is an inappropriate model of their amino acid substitution relevances.

We have demonstrated that methods for adjusting scoring matrices that rely on amino acid frequencies are ineffective in emphasising frequent residues in motifs with non-standard compositions. In the corner case where BLAST compares two identical homopolymers, the score value for their match is 0. This behaviour has numerous advantages and disadvantages depending on usage. Frequently occurring residues are considered cheap and are often present in non-functional motifs. On the other hand, rare residues, such as tryptophan, are expensive and predominantly present in functional domains^27^. Consequently, matches of rare residues are typically more significant than these of common residues. In the case of a low complexity region acting as a linker between two functional domains, a decrease in scores of frequently occurring residues has a beneficial effect on discovery of biological roles of proteins through similarity^13,28^. This is because neighbouring high complexity domains may exhibit lower similarity to homologous domains than low complexity regions to non-homologous motifs. Therefore, reducing the score of common residues may enable homology search methods to identify more similarities between domains with standard compositions than between fragments with non-standard compositions, which are often strikingly similar to each other, but in some cases functionally irrelevant. Nevertheless, fragments with non-standard compositions that perform important biological functions have also been identified. For example, two distinct protein sequences that contain similar methionine-rich motifs are involved in a similar function-droplets formation in a redox-regulated fashion^29^. For such types of fragments, we currently lack an effective method to adjust scoring matrices.

Due to divergence between homology based on model of amino acid importance and real residue importance in domains with non-standard compositions, it is necessary to develop new methods for scoring matrix adjustment and metrics for evaluating alignments of these domains. Protein similarity search methods focus on evaluating homology between protein sequences characterised by high amino acid diversity. However, a large part of the dark proteome are proteins containing fragments with non-standard compositions^6^. As we have already shown, the similarity of these fragments cannot be properly handled by state-of-the-art methods^16^. Therefore, in this study, we conducted the in-depth analysis of the scoring matrix adjustment. Equation (6) leads to the conclusion that the equation for calculating scoring matrices is unable to emphasise frequent residues Emphasising frequent residues is, however, crucial in domains with non-standard amino acid compositions as we have shown in the analyses of collagen-like domains. A partial solution is to skip adjusting scoring matrices, but according to the results presented in Table 1 alignments may still be suboptimal. Therefore, we need new methods for scoring matrix adjustment that will additionally reward biased residues when comparing motifs with non-standard compositions. As it has already been mentioned, such methods may lead to a positive expected score of alignments, thus e-value and bit score may provide irrelevant values. Consequently, we also need new metrics to evaluate alignments of sequences with non-standard compositions.

Some methods for low complexity and compositionally biased regions were developed to mask these regions for homology searches, but they have been replaced by methods for scoring matrix adjustment^7,9^. BLAST used the SEG method to mask low complexity regions as a solution to the large number of false positives caused by these highly similar regions. The SEG method, however, suffered from the problem of determining thresholds for identifying low complexity regions. Therefore, scientists developed the method for adjusting scoring matrices to smoothly diminish frequent residues^12^. In this method, regions with significantly lower complexities are diminished more than protein sequences with moderately low complexities. Later, methods for identifying different types of unusual complexities were developed to uncover new patterns that led to a better understanding of biological mechanisms^8,10^. Nevertheless, search methods should be based on a relative complexity to a query sequence rather than the absolute threshold defined by identification methods, since their goal is to find the most similar sequences also with similar complexities. This is because if the complexity of a query sequence is on a boundary of the identification method, then hits with a slightly higher complexity will be treated differently than hits with a slightly lower complexity, which is not expected. From Fig. 5b we can conclude that if we develop a general method to handle motifs with non-standard compositions by emphasising frequently occurring residues, we will still need to deal with tandem repeats in a different way.

In summary, our analysis shows why the standard approach to scoring matrix adjustment is inefficient for functional motifs with non-standard residue compositions and that we need new methods to handle them properly. To create such a method we need to break the gold standard consistency between target and background frequencies. Additionally, if we create this method then we need new metrics to evaluate alignments due to positive expected score of modified scoring matrices. These solutions will improve searching for similar domains with non-standard compositions, and thus help to discover their functional and structural properties.

## Supplementary Information

Supplementary Material.

## Data Availability

The data is available at 10.5281/zenodo.14265389.
