# Supplementary material for: Challenges in adjusting scoring matrices when comparing functional motifs with non-standard compositions
Source: Sci Rep. 2024 Dec 30;14:31777. doi: 10.1038/s41598-024-82548-8 (PMC11685636; doi:10.1038/s41598-024-82548-8)
Supplement: Supplementary file 1 — Supplementary Material. [file 41598_2024_82548_MOESM1_ESM.pdf]

Challenges in adjusting scoring matrices when comparing functional motifs with non-standard compositions

Supplementary material

Patryk Jarnot[1,*]

[1] Silesian University of Technology, Department of Computer Networks and Systems, Gliwice, 44-100, Poland

[*]patryk.jarnot@polsl.pl

BLOSUM62 adjusted for homopolymers of proline

	A	R	N	D	C	Q	E	G	H	I	L	K	M	F	P	S	T	W	Y	V
A	.0077	.0011	.0009	.0010	.0007	.0008	.0013	.0025	.0005	.0014	.0020	.0014	.0006	.0008	.0075	.0024	.0015	.0002	.0006	.0022
R	.0011	.0070	.0009	.0008	.0002	.0011	.0012	.0009	.0006	.0006	.0012	.0026	.0004	.0005	.0037	.0010	.0008	.0001	.0005	.0008
N	.0009	.0009	.0055	.0016	.0002	.0007	.0010	.0013	.0007	.0005	.0007	.0011	.0003	.0004	.0033	.0013	.0010	.0001	.0003	.0006
D	.0010	.0008	.0016	.0081	.0002	.0007	.0020	.0012	.0005	.0006	.0008	.0011	.0002	.0004	.0046	.0012	.0008	.0001	.0003	.0006
C	.0007	.0002	.0002	.0002	.0047	.0002	.0002	.0004	.0001	.0006	.0008	.0002	.0002	.0003	.0015	.0005	.0004	.0001	.0002	.0007
Q	.0008	.0011	.0007	.0007	.0002	.0027	.0014	.0006	.0005	.0004	.0008	.0013	.0003	.0003	.0031	.0008	.0006	.0001	.0003	.0005
E	.0013	.0012	.0010	.0020	.0002	.0014	.0059	.0009	.0006	.0006	.0010	.0017	.0003	.0004	.0051	.0012	.0009	.0001	.0004	.0008
G	.0025	.0009	.0013	.0012	.0004	.0006	.0009	.0152	.0005	.0007	.0011	.0012	.0004	.0006	.0053	.0017	.0010	.0002	.0004	.0009
H	.0005	.0006	.0007	.0005	.0001	.0005	.0006	.0005	.0035	.0003	.0005	.0005	.0002	.0004	.0019	.0005	.0003	.0001	.0007	.0003
I	.0014	.0006	.0005	.0006	.0006	.0004	.0006	.0007	.0003	.0077	.0051	.0007	.0011	.0015	.0040	.0008	.0012	.0002	.0007	.0051
L	.0020	.0012	.0007	.0008	.0008	.0008	.0010	.0011	.0005	.0051	.0156	.0012	.0022	.0026	.0056	.0011	.0015	.0004	.0011	.0043
K	.0014	.0026	.0011	.0011	.0002	.0013	.0017	.0012	.0005	.0007	.0012	.0059	.0004	.0005	.0056	.0013	.0010	.0001	.0005	.0009
M	.0006	.0004	.0003	.0002	.0002	.0003	.0003	.0004	.0002	.0011	.0022	.0004	.0016	.0006	.0016	.0004	.0004	.0001	.0003	.0010
F	.0008	.0005	.0004	.0004	.0003	.0003	.0004	.0006	.0004	.0015	.0026	.0005	.0006	.0076	.0022	.0006	.0006	.0004	.0019	.0013
P	.0075	.0037	.0033	.0046	.0015	.0031	.0051	.0053	.0019	.0040	.0056	.0056	.0016	.0022	.4463	.0058	.0048	.0006	.0018	.0048
S	.0024	.0010	.0013	.0012	.0005	.0008	.0012	.0017	.0005	.0008	.0011	.0013	.0004	.0006	.0058	.0045	.0018	.0001	.0005	.0011
T	.0015	.0008	.0010	.0008	.0004	.0006	.0009	.0010	.0003	.0012	.0015	.0010	.0004	.0006	.0048	.0018	.0045	.0001	.0004	.0016
W	.0002	.0001	.0001	.0001	.0001	.0001	.0001	.0002	.0001	.0002	.0004	.0001	.0001	.0004	.0006	.0001	.0001	.0026	.0004	.0002
Y	.0006	.0005	.0003	.0003	.0002	.0003	.0004	.0004	.0007	.0007	.0011	.0005	.0003	.0019	.0018	.0005	.0004	.0004	.0041	.0007
V	.0022	.0008	.0006	.0006	.0007	.0005	.0008	.0009	.0003	.0051	.0043	.0009	.0010	.0013	.0048	.0011	.0016	.0002	.0007	.0080

Figure S1. Target frequency matrix is inconsistent with background frequencies of query and hit sequences. This matrix was adjusted to background frequencies of two homopolymers of proline, both 20 residues long. Source matrix was BLOSUM62.

	A	R	N	D	C	Q	E	G	H	I	L	K	M	F	P	S	T	W	Y	V
A	5	0	0	0	1	1	1	2	0	0	0	1	1	0	-3	2	1	-1	0	1
R	0	7	1	0	-1	3	2	0	2	-1	0	4	0	-1	-4	1	1	0	0	-1
N	0	1	7	3	-1	2	1	1	2	-1	-1	1	0	-1	-4	2	2	-1	0	-1
D	0	0	3	7	-1	1	3	1	1	-1	-2	1	-1	-1	-3	1	1	-2	-1	-1
C	1	-1	-1	-1	10	-1	-2	0	-1	1	1	-1	1	0	-4	1	1	0	0	1
Q	1	3	2	1	-1	6	3	0	2	-1	0	3	1	-1	-3	1	1	0	0	0
E	1	2	1	3	-2	3	6	0	2	-1	-1	2	0	-1	-3	1	1	-1	0	-1
G	2	0	1	1	0	0	0	7	0	-2	-2	0	-1	-1	-4	1	0	0	-1	-1
H	0	2	2	1	-1	2	2	0	9	-1	-1	1	0	1	-4	1	0	0	3	-1
I	0	-1	-1	-1	1	-1	-1	-2	-1	5	3	-1	3	2	-4	-1	1	0	1	4
L	0	0	-1	-2	1	0	-1	-2	-1	3	5	-1	4	2	-4	-1	1	1	1	2
K	1	4	1	1	-1	3	2	0	1	-1	-1	6	0	-1	-3	1	1	-1	0	0
M	1	0	0	-1	1	1	0	-1	0	3	4	0	7	2	-4	0	1	1	1	2
F	0	-1	-1	-1	0	-1	-1	-1	1	2	2	-1	2	8	-5	-1	0	3	5	1
P	-3	-4	-4	-3	-4	-3	-3	-4	-4	-4	-4	-3	-4	-5	1	-3	-3	-5	-4	-4
S	2	1	2	1	1	1	1	1	1	-1	-1	1	0	-1	-3	5	3	-1	0	0
T	1	1	2	1	1	1	1	0	0	1	1	1	1	0	-3	3	6	0	0	2
W	-1	0	-1	-2	0	0	-1	0	0	0	1	-1	1	3	-5	-1	0	12	4	-1
Y	0	0	0	-1	0	0	0	-1	3	1	1	0	1	5	-4	0	0	4	8	1
V	1	-1	-1	-1	1	0	-1	-1	-1	4	2	0	2	1	-4	0	2	-1	1	5

Figure S2. Scoring matrix of potentially corrupted case. This matrix was adjusted by BLAST for homopolymeric sequences of proline, each 20 residues long. Value ln(2)/2 was used as lambda parameter for calculation. Source matrix was BLOSUM62.

In this section, we presented the resulting matrices adjusted for two homopolymers of proline using the BLAST method. These homopolymers were both 20 amino acids long. Figure S1 shows the target frequency matrix. This matrix is inconsistent with the background frequencies of poly-P sequences since it was also adjusted using pseudocounts, as described in the main text. Nevertheless, it has a high match value of proline, which is about 0.45, while the rest of the frequencies are below 0.02.

The target frequency matrix was used to calculate the scoring matrix shown in Figure S2. In this matrix, proline has the lowest score on the diagonal, which is 1. This score is lower than in the original matrix by 6. The rest of the scores on the diagonal were higher than in BLOSUM62. The mismatch values for proline decreased and were on average about -3.7 in the adjusted matrix while in the original matrix the value is approximately -2.1. Therefore, according to this matrix proline is less important than other residues.

Parameter optimisation – collagen

To optimise the BLAST parameters for the analysis, we used grid search. We searched for scoring matrices, word size, gap open and gap extend. As scoring matrices we used BLOSUM62 and PAM30. As word size we used the values 2, 3, 4 and 5. Gap open and gap extend depend on the scoring matrix chosen. Therefore, for BLOSUM we used the following pairs of gap open and gap extend: (11, 2), (10, 2), (9, 2), (8, 2), (7, 2), (6, 2), (13, 1), (12, 1), (11, 1), (10, 1), (9, 1). For PAM matrix the pairs were: (7, 2), (6, 2), (5, 2), (10, 1), (9, 1), (8, 1), (15, 3), (14, 2), (14, 1), (13, 3). We used the true positive rate as an optimisation criterion. As additional columns we also calculated alignment accuracy, alignment length and alignment count.

From the results we selected parameters for the main analysis and drew some insights on how to adjust parameters for functional motifs with non-standard compositions. In the data we can see that alignment accuracy is negatively correlated with alignment length, with the Pearson correlation coefficient of approximately -0.64. This has been discussed in the main text and shown in Fig. 5, which explains why short alignments were the cause of many false positives with high collagen alignment accuracy. All results calculated with scoring matrix adjustment had a significantly lower true positive rate than those calculated without it. In addition, all average alignment lengths in results calculated with scoring matrix adjustment were lower than results with the same alignment parameters but without scoring matrix adjustment. In Table S1 we can see for disabled adjustment BLOSUM62 scoring matrix had the highest true positive rate. However, with the adjustment enabled, the scoring matrices had comparable results. Therefore, we selected BLOSUM62 as the scoring matrix for analysis with the best alignment parameters for both enabled and disabled scoring matrix adjustment. From the results we can also infer that higher gap open penalties resulted in a higher true positive rate. For BLOSUM62 and the scoring matrix adjustment turned off, which gave the highest true positive rates, the Pearson correlation was about 0.80. This is rational as the gap open penalty in an alignment leads to irregular glycine repeats. Nevertheless, this advice apply only for motifs containing repetitions.

Table S1. Resulting statistics for alignment related parameters. Highlighted parameters were selected for analysis. These statistics were calculated for collagen-like domains.

scoring matrix	word size	gap open	gap extend	adjustment	alignment accuracy	alignment length	true positive rate	alignment count
BLOSUM62	2	13	1	FALSE	0.953	68.57	0.942	362000
BLOSUM62	4	13	1	FALSE	0.953	68.57	0.942	362000
BLOSUM62	3	13	1	FALSE	0.953	68.57	0.942	362000
BLOSUM62	5	13	1	FALSE	0.952	68.57	0.942	362000

BLOSUM62	2	11	2	FALSE	0.973	66.71	0.942	362000
BLOSUM62	3	11	2	FALSE	0.973	66.71	0.942	362000
BLOSUM62	4	11	2	FALSE	0.973	66.71	0.942	362000
BLOSUM62	2	12	1	FALSE	0.946	69.07	0.942	362000
BLOSUM62	4	12	1	FALSE	0.946	69.07	0.942	362000
BLOSUM62	3	12	1	FALSE	0.946	69.07	0.942	362000
BLOSUM62	5	11	2	FALSE	0.973	66.70	0.942	362000
BLOSUM62	2	10	2	FALSE	0.970	66.94	0.941	362000
BLOSUM62	5	12	1	FALSE	0.946	69.07	0.941	362000
BLOSUM62	3	10	2	FALSE	0.970	66.94	0.941	362000
BLOSUM62	4	10	2	FALSE	0.970	66.94	0.941	362000
BLOSUM62	5	10	2	FALSE	0.970	66.93	0.941	362000
BLOSUM62	2	11	1	FALSE	0.940	69.62	0.941	362000
BLOSUM62	3	11	1	FALSE	0.940	69.63	0.941	362000
BLOSUM62	2	9	2	FALSE	0.966	67.21	0.941	362000
BLOSUM62	4	11	1	FALSE	0.940	69.62	0.941	362000
BLOSUM62	3	9	2	FALSE	0.966	67.21	0.941	362000
BLOSUM62	4	9	2	FALSE	0.966	67.21	0.941	362000
BLOSUM62	5	11	1	FALSE	0.940	69.61	0.940	362000
BLOSUM62	5	9	2	FALSE	0.966	67.20	0.940	362000
BLOSUM62	2	8	2	FALSE	0.962	67.54	0.940	362000
BLOSUM62	3	8	2	FALSE	0.962	67.54	0.940	362000
BLOSUM62	4	8	2	FALSE	0.962	67.54	0.940	362000
BLOSUM62	5	8	2	FALSE	0.962	67.53	0.939	362000
BLOSUM62	2	10	1	FALSE	0.932	70.27	0.939	362000
BLOSUM62	3	10	1	FALSE	0.932	70.27	0.939	362000
BLOSUM62	4	10	1	FALSE	0.932	70.27	0.939	362000
BLOSUM62	5	10	1	FALSE	0.932	70.26	0.939	362000
BLOSUM62	2	7	2	FALSE	0.957	67.93	0.937	362000
BLOSUM62	3	7	2	FALSE	0.957	67.93	0.937	362000
BLOSUM62	4	7	2	FALSE	0.957	67.94	0.937	362000
BLOSUM62	5	7	2	FALSE	0.957	67.92	0.937	362000
BLOSUM62	2	9	1	FALSE	0.923	71.00	0.935	362000
BLOSUM62	3	9	1	FALSE	0.923	71.00	0.935	362000
BLOSUM62	4	9	1	FALSE	0.923	71.00	0.935	362000
BLOSUM62	5	9	1	FALSE	0.923	70.99	0.935	361998
BLOSUM62	2	6	2	FALSE	0.950	68.45	0.934	362000
BLOSUM62	3	6	2	FALSE	0.950	68.45	0.934	362000
BLOSUM62	4	6	2	FALSE	0.950	68.45	0.934	362000
BLOSUM62	5	6	2	FALSE	0.950	68.43	0.934	362000
PAM30	5	8	1	FALSE	0.745	81.42	0.932	362000
PAM30	5	9	1	FALSE	0.766	78.25	0.931	362000

PAM30	4	8	1	FALSE	0.745	81.55	0.931	362000
PAM30	4	9	1	FALSE	0.766	78.34	0.931	362000
PAM30	4	10	1	FALSE	0.786	75.51	0.931	362000
PAM30	5	10	1	FALSE	0.786	75.44	0.931	362000
PAM30	2	10	1	FALSE	0.786	75.64	0.931	362000
PAM30	2	9	1	FALSE	0.766	78.47	0.931	362000
PAM30	2	8	1	FALSE	0.745	81.68	0.931	362000
PAM30	2	14	1	FALSE	0.851	67.42	0.930	362000
PAM30	4	14	1	FALSE	0.851	67.31	0.930	362000
PAM30	5	14	1	FALSE	0.850	67.23	0.930	362000
PAM30	5	5	2	FALSE	0.813	73.14	0.930	362000
PAM30	5	6	2	FALSE	0.833	70.39	0.929	362000
PAM30	3	8	1	FALSE	0.740	81.28	0.929	362000
PAM30	3	9	1	FALSE	0.761	78.07	0.929	362000
PAM30	4	6	2	FALSE	0.833	70.47	0.929	362000
PAM30	5	7	2	FALSE	0.851	68.10	0.929	362000
PAM30	3	10	1	FALSE	0.781	75.23	0.929	362000
PAM30	4	7	2	FALSE	0.850	68.24	0.928	362000
PAM30	4	5	2	FALSE	0.813	73.28	0.928	362000
PAM30	2	14	2	FALSE	0.931	58.32	0.928	362000
PAM30	2	7	2	FALSE	0.850	68.37	0.928	362000
PAM30	2	6	2	FALSE	0.832	70.67	0.928	362000
PAM30	4	14	2	FALSE	0.930	58.19	0.928	362000
PAM30	5	14	2	FALSE	0.930	57.96	0.927	362000
PAM30	2	13	3	FALSE	0.956	55.23	0.927	362000
PAM30	2	15	3	FALSE	0.964	53.85	0.927	362000
PAM30	4	13	3	FALSE	0.956	55.08	0.927	362000
PAM30	4	15	3	FALSE	0.964	53.70	0.927	362000
PAM30	2	5	2	FALSE	0.812	73.44	0.927	362000
PAM30	3	14	1	FALSE	0.844	66.87	0.927	362000
PAM30	3	6	2	FALSE	0.829	70.03	0.927	362000
PAM30	5	13	3	FALSE	0.956	54.82	0.926	362000
PAM30	5	15	3	FALSE	0.964	53.38	0.926	362000
PAM30	3	5	2	FALSE	0.809	72.84	0.926	362000
PAM30	3	7	2	FALSE	0.846	67.67	0.926	362000
PAM30	3	14	2	FALSE	0.925	57.09	0.923	362000
PAM30	3	13	3	FALSE	0.952	53.54	0.922	362000
PAM30	3	15	3	FALSE	0.960	52.07	0.921	362000
PAM30	2	15	3	TRUE	0.962	51.71	0.875	361718
PAM30	5	15	3	TRUE	0.960	50.78	0.873	361733
PAM30	2	13	3	TRUE	0.958	51.85	0.872	361715
PAM30	4	15	3	TRUE	0.960	51.20	0.872	361694

PAM30	5	13	3	TRUE	0.956	50.98	0.870	361740
PAM30	4	13	3	TRUE	0.956	51.37	0.869	361692
PAM30	2	14	2	TRUE	0.949	52.10	0.866	361716
PAM30	5	14	2	TRUE	0.948	51.37	0.866	361738
BLOSUM62	4	9	1	TRUE	0.878	61.33	0.865	362000
BLOSUM62	2	9	1	TRUE	0.878	61.30	0.864	362000
BLOSUM62	4	10	1	TRUE	0.890	60.12	0.864	362000
BLOSUM62	2	10	1	TRUE	0.890	60.10	0.864	362000
BLOSUM62	5	9	1	TRUE	0.876	60.92	0.864	361835
BLOSUM62	3	9	1	TRUE	0.877	61.28	0.864	362000
BLOSUM62	5	10	1	TRUE	0.888	59.69	0.863	361813
BLOSUM62	3	10	1	TRUE	0.889	60.07	0.863	362000
PAM30	4	14	2	TRUE	0.947	51.67	0.863	361690
BLOSUM62	4	11	1	TRUE	0.900	58.93	0.863	362000
BLOSUM62	2	11	1	TRUE	0.900	58.92	0.862	362000
BLOSUM62	3	11	1	TRUE	0.899	58.88	0.861	362000
BLOSUM62	5	11	1	TRUE	0.897	58.47	0.861	361801
BLOSUM62	4	12	1	TRUE	0.907	57.91	0.860	362000
BLOSUM62	2	12	1	TRUE	0.907	57.89	0.860	362000
PAM30	5	14	1	TRUE	0.916	54.15	0.860	361733
BLOSUM62	3	12	1	TRUE	0.906	57.85	0.859	361998
PAM30	2	14	1	TRUE	0.916	54.60	0.858	361720
BLOSUM62	5	12	1	TRUE	0.905	57.42	0.858	361795
BLOSUM62	4	13	1	TRUE	0.913	57.02	0.858	362000
BLOSUM62	2	13	1	TRUE	0.913	57.00	0.858	362000
PAM30	2	10	1	TRUE	0.881	58.06	0.857	361730
PAM30	5	10	1	TRUE	0.881	57.54	0.856	361754
PAM30	4	14	1	TRUE	0.915	54.31	0.856	361695
BLOSUM62	3	13	1	TRUE	0.912	56.97	0.856	362000
PAM30	2	9	1	TRUE	0.869	59.36	0.856	361725
PAM30	5	9	1	TRUE	0.869	58.76	0.856	361763
BLOSUM62	5	13	1	TRUE	0.911	56.52	0.855	361791
PAM30	4	10	1	TRUE	0.881	57.75	0.855	361695
PAM30	4	9	1	TRUE	0.869	59.00	0.855	361704
PAM30	5	8	1	TRUE	0.855	60.25	0.854	361774
PAM30	4	8	1	TRUE	0.854	60.53	0.853	361713
PAM30	2	8	1	TRUE	0.853	60.93	0.853	361739
BLOSUM62	4	9	2	TRUE	0.931	54.11	0.847	362000
BLOSUM62	4	8	2	TRUE	0.925	54.75	0.847	362000
BLOSUM62	2	9	2	TRUE	0.931	54.09	0.847	362000
BLOSUM62	2	8	2	TRUE	0.925	54.74	0.847	362000
BLOSUM62	4	10	2	TRUE	0.935	53.58	0.846	362000

BLOSUM62	4	7	2	TRUE	0.918	55.55	0.846	362000
BLOSUM62	2	10	2	TRUE	0.934	53.57	0.846	362000
BLOSUM62	2	7	2	TRUE	0.918	55.53	0.846	362000
PAM30	5	7	2	TRUE	0.908	53.58	0.846	361757
BLOSUM62	3	8	2	TRUE	0.924	54.72	0.846	361998
BLOSUM62	3	9	2	TRUE	0.930	54.06	0.845	361995
BLOSUM62	4	11	2	TRUE	0.937	53.17	0.845	362000
BLOSUM62	5	8	2	TRUE	0.922	54.19	0.845	361802
BLOSUM62	2	11	2	TRUE	0.937	53.15	0.845	362000
PAM30	2	7	2	TRUE	0.906	54.22	0.844	361721
BLOSUM62	3	7	2	TRUE	0.917	55.51	0.844	362000
BLOSUM62	3	10	2	TRUE	0.933	53.54	0.844	361996
PAM30	4	7	2	TRUE	0.906	53.89	0.844	361690
BLOSUM62	5	7	2	TRUE	0.915	54.99	0.844	361819
BLOSUM62	5	9	2	TRUE	0.927	53.52	0.844	361794
BLOSUM62	3	11	2	TRUE	0.936	53.12	0.843	361998
BLOSUM62	5	10	2	TRUE	0.931	52.99	0.843	361787
BLOSUM62	4	6	2	TRUE	0.907	56.38	0.842	362000
BLOSUM62	2	6	2	TRUE	0.907	56.37	0.842	362000
BLOSUM62	5	11	2	TRUE	0.934	52.56	0.841	361786
BLOSUM62	5	6	2	TRUE	0.904	55.85	0.841	361838
BLOSUM62	3	6	2	TRUE	0.906	56.35	0.841	362000
PAM30	5	6	2	TRUE	0.896	54.33	0.840	361760
PAM30	4	6	2	TRUE	0.895	54.68	0.839	361689
PAM30	2	6	2	TRUE	0.895	55.06	0.839	361724
PAM30	5	5	2	TRUE	0.880	55.39	0.832	361781
PAM30	4	5	2	TRUE	0.879	55.84	0.831	361693
PAM30	2	5	2	TRUE	0.877	56.20	0.827	361732
PAM30	3	15	3	TRUE	0.931	47.20	0.806	361837
PAM30	3	13	3	TRUE	0.927	47.49	0.802	361837
PAM30	3	9	1	TRUE	0.841	56.96	0.801	361831
PAM30	3	8	1	TRUE	0.825	58.73	0.800	361821
PAM30	3	10	1	TRUE	0.853	55.48	0.800	361824
PAM30	3	14	1	TRUE	0.888	51.54	0.799	361835
PAM30	3	14	2	TRUE	0.918	47.99	0.798	361836
PAM30	3	7	2	TRUE	0.875	51.05	0.779	361817
PAM30	3	6	2	TRUE	0.862	52.13	0.774	361820
PAM30	3	5	2	TRUE	0.844	53.64	0.765	361817

Coiled coil analysis

Introduction

In this work, we stated that frequently occurring residues play key roles in functional motifs with non-standard compositions, and therefore diminishing them in a scoring matrix has a negative effect on searching for similar domains. We have already shown this in the analysis of collagen-like domains. In this section, we have tested whether this statement can also be applied to coiled coil domains in order to strengthen the main conclusions of the manuscript.

Methods

First, we downloaded all proteins containing coiled coils from UniProtKB/Swiss-Prot in txt format. The UniProtKB query was "(ft_coiled:*) AND (reviewed:true)". There were 15,660 such proteins in the database. From each record in the txt file, we read a UniProt AC, the positions of coiled coil domains and a protein sequence. We then selected these domains in which at least one residue had a frequency greater than 0.333 – a similar threshold to that used for collagen. Using this procedure, we obtained 1012 coiled coils with non-standard compositions. We further used these domains as queries to search for similarities with the scoring matrix turned on and off. Moreover, we used grid search to find the best set of alignment parameters. For the searches we used e-value of 10,000,000 and limited the number of output alignments to 50. This was done to obtain a comparable number of results and thus be able to compare true positive rates.

Results

Table S2 presents the true positive rates for all sets of parameters. For parameter optimisation, we changed the scoring matrix, word size, gap open, gap extend and scoring matrix adjustment in the same way as for the collagen analysis. For all parameter sets, the true positive rate was higher when scoring matrix adjustment was disabled than when it was enabled. Minimal difference was about 0.010, while maximal was about 0.075. The average difference was approximately 0.042 with a standard deviation of about 0.021. For the best parameter sets the difference was also about 0.021.

Conclusions

The results of this analysis confirm that functions of domains with non-standard compositions rely on frequently occurring residues, and therefore diminishing them in a scoring matrix decreases the true positive rate. Consequently, it supports the conclusions drawn from the analysis of collagen-like domains.

Table S2. Resulting statistics for alignment related parameters. Highlighted parameters were selected for analysis. These statistics were calculated for coiled coils.

scoring matrix	word size	gap open	gap extend	adjustment	true positive rate	alignment count
PAM30	2	8	1	FALSE	0.347	50600
BLOSUM62	2	11	1	FALSE	0.347	50600
BLOSUM62	4	11	1	FALSE	0.347	50600
BLOSUM62	2	12	1	FALSE	0.345	50600
PAM30	4	8	1	FALSE	0.345	50600
BLOSUM62	4	12	1	FALSE	0.345	50600

BLOSUM62	3	11	1	FALSE	0.344	50600
BLOSUM62	4	8	2	FALSE	0.344	50600
BLOSUM62	2	8	2	FALSE	0.344	50600
BLOSUM62	4	10	1	FALSE	0.344	50600
BLOSUM62	2	10	1	FALSE	0.344	50600
BLOSUM62	3	12	1	FALSE	0.344	50600
PAM30	3	8	1	FALSE	0.344	50600
BLOSUM62	3	8	2	FALSE	0.343	50600
BLOSUM62	3	10	1	FALSE	0.342	50600
BLOSUM62	2	13	1	FALSE	0.342	50600
BLOSUM62	4	13	1	FALSE	0.342	50600
PAM30	2	5	2	FALSE	0.342	50600
PAM30	2	9	1	FALSE	0.342	50600
PAM30	4	5	2	FALSE	0.341	50600
PAM30	5	8	1	FALSE	0.341	50600
PAM30	4	9	1	FALSE	0.341	50600
BLOSUM62	3	13	1	FALSE	0.341	50600
BLOSUM62	4	7	2	FALSE	0.340	50600
PAM30	3	5	2	FALSE	0.340	50600
BLOSUM62	2	7	2	FALSE	0.340	50600
PAM30	2	6	2	FALSE	0.340	50600
BLOSUM62	2	9	2	FALSE	0.340	50600
BLOSUM62	2	11	2	FALSE	0.340	50600
PAM30	3	9	1	FALSE	0.340	50600
BLOSUM62	4	9	2	FALSE	0.340	50600
BLOSUM62	3	7	2	FALSE	0.340	50600
BLOSUM62	3	9	2	FALSE	0.340	50600
BLOSUM62	4	11	2	FALSE	0.339	50600
BLOSUM62	2	10	2	FALSE	0.339	50600
PAM30	4	6	2	FALSE	0.339	50600
BLOSUM62	4	10	2	FALSE	0.339	50600
BLOSUM62	3	11	2	FALSE	0.339	50600
BLOSUM62	3	10	2	FALSE	0.339	50600
PAM30	2	7	2	FALSE	0.338	50600
PAM30	2	10	1	FALSE	0.338	50600
PAM30	5	5	2	FALSE	0.337	50600
PAM30	4	10	1	FALSE	0.337	50600
PAM30	3	6	2	FALSE	0.337	50600
PAM30	5	9	1	FALSE	0.337	50600
PAM30	4	7	2	FALSE	0.336	50600
BLOSUM62	2	6	2	FALSE	0.336	50600
BLOSUM62	5	12	1	FALSE	0.336	48072

BLOSUM62	5	11	1	FALSE	0.336	48071
BLOSUM62	5	10	1	FALSE	0.336	48071
BLOSUM62	5	13	1	FALSE	0.336	48072
PAM30	3	10	1	FALSE	0.336	50600
BLOSUM62	5	8	2	FALSE	0.336	48072
BLOSUM62	4	6	2	FALSE	0.336	50600
BLOSUM62	3	6	2	FALSE	0.335	50600
PAM30	5	6	2	FALSE	0.335	50600
BLOSUM62	2	9	1	FALSE	0.335	50600
BLOSUM62	4	9	1	FALSE	0.335	50600
PAM30	3	7	2	FALSE	0.334	50600
BLOSUM62	5	7	2	FALSE	0.334	48071
BLOSUM62	5	9	2	FALSE	0.334	48072
BLOSUM62	5	11	2	FALSE	0.334	48072
BLOSUM62	3	9	1	FALSE	0.334	50600
BLOSUM62	5	10	2	FALSE	0.333	48072
PAM30	5	7	2	FALSE	0.333	50600
PAM30	5	10	1	FALSE	0.332	50600
BLOSUM62	5	6	2	FALSE	0.331	48071
BLOSUM62	5	9	1	FALSE	0.330	48062
PAM30	2	14	1	FALSE	0.328	50600
PAM30	4	14	1	FALSE	0.327	50600
BLOSUM62	3	8	2	TRUE	0.326	47849
PAM30	3	14	1	FALSE	0.326	50600
BLOSUM62	3	10	1	TRUE	0.326	48162
BLOSUM62	3	11	1	TRUE	0.325	48026
BLOSUM62	4	11	1	TRUE	0.325	48351
BLOSUM62	4	8	2	TRUE	0.325	48225
BLOSUM62	4	10	1	TRUE	0.324	48471
BLOSUM62	3	7	2	TRUE	0.324	47905
PAM30	5	14	1	FALSE	0.324	50600
PAM30	2	14	2	FALSE	0.324	50600
BLOSUM62	3	9	1	TRUE	0.323	48328
BLOSUM62	3	9	2	TRUE	0.323	47797
PAM30	4	14	2	FALSE	0.323	50600
BLOSUM62	3	6	2	TRUE	0.323	48260
BLOSUM62	2	10	1	TRUE	0.323	48527
PAM30	4	13	3	FALSE	0.323	50600
BLOSUM62	3	10	2	TRUE	0.322	47726
PAM30	2	13	3	FALSE	0.322	50600
BLOSUM62	4	7	2	TRUE	0.322	48232
BLOSUM62	2	11	1	TRUE	0.322	48407

BLOSUM62	3	12	1	TRUE	0.322	47854
BLOSUM62	4	9	1	TRUE	0.321	48600
PAM30	2	15	3	FALSE	0.321	50600
BLOSUM62	4	9	2	TRUE	0.321	48174
PAM30	3	14	2	FALSE	0.321	50600
BLOSUM62	2	8	2	TRUE	0.321	48296
PAM30	4	15	3	FALSE	0.321	50600
BLOSUM62	4	6	2	TRUE	0.321	48568
BLOSUM62	4	10	2	TRUE	0.320	48106
PAM30	5	14	2	FALSE	0.320	50600
BLOSUM62	4	12	1	TRUE	0.320	48189
PAM30	5	13	3	FALSE	0.320	50600
PAM30	3	13	3	FALSE	0.320	50600
BLOSUM62	3	13	1	TRUE	0.320	47839
BLOSUM62	3	11	2	TRUE	0.319	47830
BLOSUM62	2	9	1	TRUE	0.319	48636
BLOSUM62	4	13	1	TRUE	0.319	48189
PAM30	3	15	3	FALSE	0.319	50600
BLOSUM62	2	7	2	TRUE	0.319	48272
BLOSUM62	4	11	2	TRUE	0.319	48185
PAM30	5	15	3	FALSE	0.318	50600
BLOSUM62	2	6	2	TRUE	0.318	48585
BLOSUM62	2	10	2	TRUE	0.318	48180
BLOSUM62	2	12	1	TRUE	0.317	48263
BLOSUM62	2	9	2	TRUE	0.317	48242
BLOSUM62	2	11	2	TRUE	0.316	48224
BLOSUM62	2	13	1	TRUE	0.316	48246
PAM30	2	5	2	TRUE	0.299	48479
PAM30	4	5	2	TRUE	0.299	46425
PAM30	3	5	2	TRUE	0.294	46890
PAM30	5	5	2	TRUE	0.293	42405
PAM30	4	6	2	TRUE	0.292	46006
BLOSUM62	5	8	2	TRUE	0.292	40021
BLOSUM62	5	9	2	TRUE	0.291	39758
PAM30	2	8	1	TRUE	0.290	49474
BLOSUM62	5	10	2	TRUE	0.290	39397
BLOSUM62	5	13	1	TRUE	0.290	39739
BLOSUM62	5	12	1	TRUE	0.290	39887
PAM30	2	6	2	TRUE	0.289	48349
BLOSUM62	5	11	2	TRUE	0.289	39484
PAM30	4	7	2	TRUE	0.289	45455
PAM30	4	8	1	TRUE	0.288	48650

BLOSUM62	5	11	1	TRUE	0.288	40461
PAM30	3	6	2	TRUE	0.288	46716
BLOSUM62	5	7	2	TRUE	0.288	40479
BLOSUM62	5	6	2	TRUE	0.287	41754
BLOSUM62	5	10	1	TRUE	0.285	41048
PAM30	2	9	1	TRUE	0.285	49219
PAM30	5	6	2	TRUE	0.285	41288
PAM30	4	9	1	TRUE	0.284	48061
PAM30	2	7	2	TRUE	0.283	48215
PAM30	3	8	1	TRUE	0.282	48797
BLOSUM62	5	9	1	TRUE	0.282	41989
PAM30	3	7	2	TRUE	0.281	46233
PAM30	5	8	1	TRUE	0.279	46714
PAM30	5	7	2	TRUE	0.279	40328
PAM30	4	10	1	TRUE	0.278	47285
PAM30	3	9	1	TRUE	0.277	48248
PAM30	2	10	1	TRUE	0.276	48916
PAM30	5	9	1	TRUE	0.274	45206
PAM30	3	10	1	TRUE	0.272	47673
PAM30	5	10	1	TRUE	0.268	43684
PAM30	4	14	1	TRUE	0.260	45297
PAM30	3	14	1	TRUE	0.256	45938
PAM30	2	14	1	TRUE	0.256	48228
PAM30	5	14	1	TRUE	0.256	39612
PAM30	4	14	2	TRUE	0.255	44639
PAM30	4	13	3	TRUE	0.255	44695
PAM30	5	14	2	TRUE	0.254	38093
PAM30	5	13	3	TRUE	0.254	38143
PAM30	4	15	3	TRUE	0.253	44674
PAM30	5	15	3	TRUE	0.253	38042
PAM30	3	14	2	TRUE	0.253	45242
PAM30	3	13	3	TRUE	0.251	45213
PAM30	2	14	2	TRUE	0.250	48111
PAM30	2	13	3	TRUE	0.249	48128
PAM30	3	15	3	TRUE	0.249	45190
PAM30	2	15	3	TRUE	0.247	48110
